# Management of Renal Stones >2 cm in the Modern Endourology Era: Does PCNL Remain the Gold Standard?

**DOI:** 10.3390/jcm15207830

**Published:** 2026-10-10

**Authors:** Nidal Samira, Murad Asali

**Affiliations:** Urology Department, Barzilai Medical Center, Ben-Gurion University of the Negev, Ashkelon 8410501, Israel; dr.muradasali@gmail.com

**Keywords:** mini-PCNL, suction ureteral access sheath, intrarenal pressure, stone-free rate

## Abstract

**Background:** Percutaneous nephrolithotomy (PCNL) remains the reference treatment for renal stones larger than 2 cm because of its high single-session stone-free rate. However, advances in flexible ureteroscopy (fURS), laser lithotripsy, suction-assisted access systems, and endoscopic technology have expanded the role of retrograde intrarenal surgery (RIRS) in patients who historically would have been treated primarily with PCNL. **Objective**: To critically review the contemporary roles of PCNL and RIRS in the management of renal stones >2 cm and evaluate whether advances in endoscopic and laser technologies have altered the traditional treatment paradigm. **Methods:** A focused narrative review of the contemporary literature was performed using PubMed, Scopus, and Web of Science. Priority was given to randomized trials, prospective comparative studies, multicenter registries, systematic reviews, meta-analyses, and international guideline recommendations evaluating efficacy, safety, retreatment, operative burden, and technological developments relevant to the management of large renal stones. **Results:** PCNL continues to provide the most reliable single-session stone clearance for large and complex renal calculi, particularly in patients with high stone burden and staghorn disease. RIRS offers lower procedural morbidity and faster recovery but may require staged procedures as stone burden increases. Recent developments, including thulium fiber laser, pulse-modulated Holmium:YAG systems, suction-assisted ureteral access sheaths, improved digital ureteroscopy, and intrarenal pressure-control strategies, have increased the efficiency and potential applicability of RIRS for larger stones. Nevertheless, current evidence does not support replacing PCNL as the reference treatment for complex or high-volume stone disease. **Conclusion:** PCNL remains the reference standard for renal stones >2 cm when maximal single-session clearance is the principal objective. Modern RIRS increasingly represents a clinically relevant alternative in appropriately selected patients. Rather than defining a single superior procedure, contemporary management should integrate stone burden and complexity, patient comorbidity, treatment goals, available technology, and local expertise.

## 1. Introduction

Percutaneous nephrolithotomy (PCNL) remains the reference treatment for large renal calculi and is recommended as first-line therapy for renal stones larger than 2 cm [1]. Its principal advantage is the ability to achieve high stone-free rates (SFRs) and effective clearance of substantial or complex stone burdens, frequently within a single treatment episode. However, this efficacy must be balanced against the morbidity inherent to percutaneous renal access, particularly bleeding, postoperative pain, and hospitalization [1,2].

For clarity, this review distinguishes four procedural approaches. Standard PCNL involves percutaneous renal access using a conventional-sized tract, whereas mini-PCNL uses a smaller access tract intended to reduce access-related morbidity while preserving the principles of percutaneous stone removal. Conventional RIRS involves flexible ureteroscopic access to the collecting system, laser lithotripsy, and fragment management without an integrated active-suction access system. Suction-assisted RIRS incorporates active fragment evacuation through a suction-enabled access device; the flexible and navigable suction ureteral access sheath (FANS) is one such system. These approaches should not be treated as interchangeable because tract size, access route, fragment evacuation, and associated morbidity differ. In particular, findings from trials comparing RIRS with mini-PCNL cannot automatically be generalized to standard PCNL.

Over the past decade, the boundaries of retrograde intrarenal surgery (RIRS) have expanded considerably. Improvements in flexible ureteroscope design, digital visualization, ureteral access sheaths, laser lithotripsy, irrigation systems, and perioperative management have enabled increasingly complex renal stones to be approached through a retrograde route. Nevertheless, contemporary randomized evidence comparing RIRS with miniaturized PCNL indicates that mini-PCNL generally retains an advantage in single-session stone clearance, whereas RIRS is associated with reduced bleeding and shorter hospitalization [2].

The evolution of laser technology has contributed substantially to the increasing efficiency of RIRS. Holmium:YAG (Ho:YAG) remains an established lithotripsy platform, while thulium fiber laser (TFL) has emerged as an alternative with favorable technical characteristics for endoscopic stone treatment. Randomized clinical evidence has demonstrated effective ureteroscopic lithotripsy with both technologies, with differences in specific operative and stone-clearance outcomes [3]. Importantly, TFL and Ho:YAG should not be considered independent alternatives to PCNL; rather, they are lithotripsy technologies used within endourological procedures and may influence the efficiency and performance of RIRS.

More recently, suction-assisted ureteral access systems have addressed important limitations of conventional RIRS, particularly fragment evacuation and maintenance of endoscopic visualization. Flexible and navigable suction ureteral access sheaths (FANS) combine flexible access with active suction, facilitating simultaneous irrigation and evacuation of stone fragments. Evidence supporting this approach has expanded rapidly. An international multicenter randomized noninferiority trial involving 720 patients with 2–3 cm renal stones directly compared FANS-assisted flexible ureteroscopy with mini-PCNL [4]. Subsequent randomized evidence and meta-analysis have suggested that final stone-free outcomes may be comparable between FANS-RIRS and mini-PCNL in appropriately selected 2–3 cm stones, while FANS-RIRS may reduce blood loss, postoperative pain, and length of hospitalization [5,6].

These developments have shifted the contemporary debate away from a simple comparison between PCNL and conventional flexible ureteroscopy. The more relevant clinical question is whether modern RIRS—supported by improved laser lithotripsy, suction-assisted access, enhanced visualization, and strategies for optimizing irrigation and intrarenal pressure—can provide an effective alternative to PCNL in appropriately selected patients with renal stones larger than 2 cm.

This review therefore examines the contemporary roles of PCNL and RIRS in the management of renal stones >2 cm. Particular emphasis is placed on stone-free outcomes, need for staged or auxiliary procedures, perioperative morbidity, hospital stay, and patient selection, together with the impact of modern laser and suction technologies. The objective is not to identify a universally superior procedure, but to define the clinical circumstances in which PCNL continues to offer a clear advantage and those in which technological advances may justify an expanded role for RIRS.

## 2. Methods

### 2.1. Review Design

This article was designed as a focused narrative review of contemporary evidence regarding the management of renal stones larger than 2 cm. The review aimed to evaluate the current roles of percutaneous nephrolithotomy (PCNL) and retrograde intrarenal surgery (RIRS), with particular attention to technological developments that may influence the relative efficacy, safety, and clinical applicability of these approaches.

### 2.2. Literature Search

A focused literature search was conducted using PubMed/MEDLINE, Scopus, and Web of Science for publications from January 2020 through September 2026, with the search last updated in September 2026. The search was restricted to English-language publications and focused on PCNL, miniaturized PCNL, flexible ureteroscopy, RIRS, laser lithotripsy, and suction-assisted ureteroscopic technologies relevant to the management of large renal stones. Earlier landmark studies were considered selectively when required to provide relevant historical or technological context.

Search concepts included combinations of terms related to “renal stones,” “kidney stones,” “large renal stones,” “>2 cm,” “2–3 cm,” “percutaneous nephrolithotomy,” “PCNL,” “mini-PCNL,” “flexible ureteroscopy,” “retrograde intrarenal surgery,” “RIRS,” “thulium fiber laser,” “Holmium:YAG,” “suction ureteral access sheath,” and “flexible and navigable suction ureteral access sheath.”

Studies were selected according to their relevance to the contemporary management of renal stones >2 cm. Priority was given to randomized controlled trials, prospective comparative studies, multicenter studies and registries, systematic reviews and meta-analyses, and international clinical practice guidelines. Studies directly comparing PCNL or mini-PCNL with conventional or suction-assisted RIRS were prioritized. Studies involving smaller stones were included selectively when they provided relevant evidence regarding laser technology, suction systems, intrarenal pressure, or other technological developments applicable to contemporary RIRS. Case reports, small case series, conference abstracts without sufficient outcome data, and publications without direct relevance to the clinical or technological questions addressed in this review were excluded from the principal evidence synthesis. Because this was a narrative review, study selection was relevance-based, and no formal PRISMA screening process or risk-of-bias assessment was undertaken. 

### 2.3. Evidence Selection and Synthesis

Because the objective of this review was interpretive rather than to re-pool existing evidence, effect estimates from published meta-analyses were reported only as results of the respective source publications. No de novo pooling, reweighting, or cross-meta-analysis quantitative synthesis was performed. A formal umbrella-review framework was therefore not adopted. Importantly, where primary-study overlap existed between meta-analyses, these meta-analyses were considered overlapping rather than independent evidence and were not interpreted as separate confirmation of an effect.

Evidence was selected according to its relevance to the contemporary management of renal stones >2 cm, with particular emphasis on studies directly comparing percutaneous and retrograde approaches or evaluating technologies capable of influencing these treatment strategies. Studies involving smaller stones were considered selectively when they provided clinically relevant evidence regarding laser performance, suction technology, procedural safety, or other technological developments applicable to RIRS.

The principal outcomes considered were stone-free rate, single-session stone clearance, requirement for staged or auxiliary procedures, operative time, perioperative complications, bleeding and transfusion, postoperative pain, length of hospital stay, and retreatment. Where available, differences in stone-free definitions, imaging modalities, follow-up intervals, and procedural techniques were considered when interpreting comparisons between studies.

The literature was synthesized qualitatively rather than quantitatively because of heterogeneity in study design, patient selection, stone characteristics, definitions of stone-free status, imaging protocols, and surgical techniques. Particular emphasis was placed on the clinical interpretation of the evidence and on identifying circumstances in which PCNL remains advantageous and those in which contemporary RIRS may represent a reasonable alternative.

### 2.4. Scope of the Review

The review was not intended as a systematic review or meta-analysis, and no formal pooled effect estimates or risk-of-bias assessment were performed. Instead, the objective was to provide a clinically oriented synthesis of contemporary evidence and emerging technologies relevant to treatment selection for renal stones larger than 2 cm.

## 3. Results: Contemporary Evidence

### 3.1. PCNL as the Reference Standard for Large Renal Stones

Percutaneous nephrolithotomy (PCNL) remains the reference surgical treatment for renal stones larger than 2 cm. Current European Association of Urology guidelines recommend PCNL as first-line treatment for stones >2 cm, while flexible ureteroscopy may be considered when PCNL is not an appropriate option [1]. The principal advantage of PCNL is its ability to achieve effective stone clearance in a single treatment session, particularly in patients with high stone burden, multiple calculi, complex collecting-system anatomy, or partial and complete staghorn stones.

Miniaturization of percutaneous access has sought to preserve the efficacy of PCNL while reducing access-related morbidity. Mini-PCNL has consequently become particularly relevant when comparing percutaneous and retrograde approaches for intermediate and large renal stone burdens.

The distinction between standard and mini-PCNL is clinically relevant when interpreting contemporary comparative evidence. In a randomized study of 80 patients with 2–4 cm renal stones, Wishahi et al. reported comparable stone-free rates with mini-PCNL and standard PCNL (80% vs. 85%, respectively), while mini-PCNL was associated with longer operative time but reduced perioperative morbidity and shorter hospitalization [7]. These findings illustrate that tract miniaturization may modify the morbidity profile of percutaneous treatment without necessarily compromising stone clearance and reinforce the need to distinguish mini-PCNL from standard PCNL when comparing percutaneous approaches with RIRS.

Contemporary PCNL has also evolved through the incorporation of suction- and vacuum-assisted systems, particularly in mini-PCNL. These systems combine percutaneous access with active aspiration of irrigation fluid and stone fragments, potentially facilitating fragment evacuation, improving endoscopic visualization, and limiting intrarenal pressure. A recent meta-analysis of 14 comparative studies including 2510 patients found that vacuum-assisted access sheath mini-PCNL was associated with shorter operative time, higher stone-free rates, lower postoperative fever and sepsis rates, fewer overall complications, and shorter hospitalization compared with conventional access sheath mini-PCNL, although no significant differences were observed in transfusion rate or hemoglobin reduction. These findings indicate that technological evolution in contemporary stone surgery is not limited to RIRS and that suction-assisted mini-PCNL represents an important development within the percutaneous approach [8]. 

Importantly, the pivotal randomized comparisons of FANS-assisted RIRS with mini-PCNL did not clearly characterize the percutaneous comparator as a suction-assisted mini-PCNL technique; therefore, their findings should not be interpreted as direct comparisons between suction-assisted RIRS and suction-assisted mini-PCNL [4,6].

A 2026 meta-analysis of 30 randomized controlled trials involving 4173 patients with renal stones predominantly measuring 1–3 cm demonstrated a significantly higher single-session stone-free rate with mini-PCNL than with RIRS (RR 0.92, 95% CI 0.88–0.96; *p* < 0.001) [2]. RIRS was associated with lower transfusion requirements, a smaller reduction in hemoglobin, and shorter hospitalization. The analysis also reported a lower point estimate for overall complications with RIRS, although this difference did not reach conventional statistical significance (RR 0.79; *p* = 0.057) [2]. Operative time and high-grade Clavien–Dindo complications were comparable, whereas RIRS required significantly more postoperative auxiliary procedures [2]. These findings illustrate the trade-off between the approaches: mini-PCNL generally achieves higher single-session clearance, while RIRS may reduce access-related morbidity. Because the included stones predominantly ranged from 1 to 3 cm, these pooled findings should not be extrapolated uncritically to all renal stones larger than 2 cm.

Additional contemporary evidence specifically examining renal stones >2 cm supports this distinction. In a systematic review and Bayesian network meta-analysis including 40 studies and 6696 patients, Hinojosa-Gonzalez et al. stratified outcomes according to stone size and found that PCNL, mini-PCNL, and ultramini-PCNL achieved higher stone-free rates than RIRS in patients with renal stones >2 cm [9]. These findings strengthen the evidence supporting percutaneous approaches for larger renal stones while also highlighting the importance of distinguishing among different PCNL tract sizes when interpreting comparative outcomes.

### 3.2. Contemporary RIRS for Large Renal Stones

The role of RIRS has expanded beyond the treatment of small and intermediate renal calculi. Improvements in flexible ureteroscope design, digital visualization, ureteral access sheaths, laser lithotripsy, irrigation systems, and perioperative pathways have made treatment of selected stones >2 cm increasingly feasible.

Nevertheless, conventional RIRS has important limitations as stone burden increases. Larger stones require prolonged fragmentation or dusting and more extensive fragment extraction, potentially increasing operative time. Residual fragments may necessitate staged ureteroscopy or auxiliary procedures. This is supported by contemporary randomized evidence showing a greater requirement for postoperative auxiliary procedures after conventional RIRS compared with mini-PCNL [2].

Accordingly, the principal limitation of RIRS for large renal stones is not simply whether fragmentation is technically possible, but whether effective clearance can be achieved efficiently and safely without multiple procedures. For patients in whom maximal single-session clearance is the overriding objective, particularly those with complex or high-volume stone disease, PCNL retains an important advantage. Conversely, RIRS becomes attractive when minimizing bleeding, postoperative morbidity, and hospitalization is particularly important [1,2].

Direct randomized evidence also supports the feasibility of RIRS in selected patients enrolled under a 2–3 cm eligibility criterion. In a prospective randomized trial of 122 patients, Kassem et al. reported comparable 1-month stone-free rates following RIRS and PNL (70.5% vs. 73.8%, respectively; *p* = 0.840), with stone-free status assessed by computed tomography [10]. However, mean stone size was 2.11 ± 0.21 cm in the RIRS group and 2.12 ± 0.23 cm in the PNL group, indicating that the study population was concentrated near the lower boundary of the 2–3 cm range. RIRS was associated with longer lithotripsy time, whereas PNL resulted in greater postoperative hemoglobin reduction. Accordingly, these findings support RIRS as a potential alternative particularly for stones just above 2 cm, but provide more limited evidence for stones approaching 3 cm.

### 3.3. Impact of Modern Laser Technology

Advances in laser lithotripsy have contributed substantially to the expansion of RIRS. Holmium:YAG (Ho:YAG) remains an established lithotripsy platform, while thulium fiber laser (TFL) has emerged as an alternative technology for endoscopic stone treatment.

In a prospective randomized clinical trial of 120 patients, Ulvik et al. compared TFL with Ho:YAG during ureteroscopic lithotripsy [3]. TFL achieved a significantly higher overall single-session stone-free rate than Ho:YAG (92% vs. 67%; *p* = 0.001). The difference was particularly pronounced for renal stones, for which the stone-free rate was 86% with TFL compared with 49% with Ho:YAG (*p* = 0.001). These findings demonstrate that advances in laser technology can meaningfully improve stone-clearance outcomes during ureteroscopy. However, this study did not compare RIRS with PCNL, included a relatively small overall cohort, and was not specifically designed to evaluate renal stones >2 cm. Therefore, the observed advantage of TFL should be interpreted as evidence of improved lithotripsy performance within ureteroscopic treatment rather than evidence that TFL-assisted RIRS achieves outcomes equivalent to PCNL for large renal stones.

The relevance of TFL to renal stones >2 cm should therefore be interpreted within the context of RIRS rather than as an independent alternative to PCNL. Improvements in fragmentation and dusting efficiency may facilitate treatment of larger stone burdens, but laser performance alone does not eliminate the other limitations of RIRS, including fragment evacuation, intrarenal pressure, operative duration, and the potential requirement for staged treatment.

Pulse modulation represents another development in contemporary Ho:YAG lithotripsy. Comparative clinical evidence evaluating Moses pulse modulation against standard Ho:YAG has demonstrated shorter lasing time and greater stone-ablation speed, although improvements in overall operative time, stone-free rate, and complication rates have not been consistently demonstrated [11]. Accordingly, pulse-modulated Ho:YAG should be considered an incremental improvement in lithotripsy efficiency rather than evidence that laser technology alone changes the relative indications for RIRS and PCNL.

### 3.4. Suction-Assisted RIRS and FANS

Fragment evacuation represents an important limitation of conventional RIRS when treating larger stone burdens. Suction-assisted ureteroscopic systems have therefore emerged as a potentially important development in endoscopic stone surgery.

Flexible and navigable suction ureteral access sheaths (FANS) combine flexible access with active suction, allowing aspiration of stone fragments during flexible ureteroscopy. The ability to evacuate fragments during lithotripsy may reduce dependence on spontaneous passage of stone dust and facilitate more complete endoscopic clearance.

An international multicenter randomized noninferiority trial enrolled 720 patients with 2–3 cm renal stones across 12 centers and compared FANS-assisted flexible ureteroscopy with mini-PCNL [4]. FANS-assisted ureteroscopy met the prespecified noninferiority criterion for the primary endpoint of immediate SFR and was associated with a lower transfusion rate and shorter postoperative hospitalization, although operative time was longer [4]. No significant differences were observed in auxiliary procedures, 3-month SFR, or infection-related complications. Nevertheless, interpretation of this trial requires consideration of subsequent methodological correspondence. Concerns raised after publication included aspects of the noninferiority design and margin, the definition and assessment of stone-free status, and the generalizability of the findings across different clinical settings. The investigators subsequently responded to these concerns and defended the methodological choices of the trial. Accordingly, the study provides important randomized evidence supporting FANS-assisted RIRS for selected 2–3 cm renal stones, but its findings should be considered within the specific trial design, patient population, outcome definitions, and available external validation rather than regarded as definitive evidence of equivalence between FANS-RIRS and mini-PCNL.

This comparable immediate clearance was achieved with different perioperative trade-offs. FANS-assisted ureteroscopy required a longer operative time, with a mean difference of 29 min, but was associated with a lower transfusion rate and a 2.5-day shorter postoperative hospitalization [4]. Quality-of-life improvement was also greater following FANS-assisted ureteroscopy. No significant differences were identified in auxiliary procedures, 3-month SFR, or infection-related complications [4].

These findings provide important evidence that the efficacy gap between retrograde and percutaneous treatment can be substantially narrowed when modern suction-assisted technology is incorporated into RIRS.

### 3.5. FANS-RIRS Versus Mini-PCNL: Accumulating Randomized Evidence

Li et al. conducted a systematic review and meta-analysis of three randomized controlled trials involving 1020 patients with 2–3 cm renal stones [5]. The pooled analysis found no statistically significant difference in final stone-free rate (SFR) between FANS-RIRS and mini-PCNL (OR 0.80, 95% CI 0.51–1.24; *p* = 0.31). Importantly, the findings of individual trials included in this meta-analysis should not be interpreted as independent confirmation of the pooled results.

FANS-RIRS was associated with a smaller decrease in hemoglobin, lower rates of hemorrhage and blood transfusion, shorter hospitalization, and less postoperative pain [5]. No statistically significant differences were observed in operative time, auxiliary procedure rates, or infectious complications. However, the certainty of evidence for several outcomes was moderate to low, reflecting the limited number of randomized trials and between-study heterogeneity.

Wang et al. subsequently reported a randomized trial involving 326 patients with 2–3 cm upper urinary tract stones, including both renal and ureteral calculi [6]. Immediate and 1-month SFRs were comparable between FANS-assisted flexible ureteroscopy and mini-PCNL. FANS-assisted ureteroscopy was associated with less hemoglobin loss and shorter hospitalization, although operative time was longer in the overall cohort. Infection-related complications did not differ significantly [6]. Because the trial included both renal and ureteral stones, its overall findings should not be interpreted as evidence derived exclusively from patients with renal calculi, which limits direct applicability to the renal-stone population considered in this review.

In the prespecified subgroup of patients with stones measuring ≥2 to <2.5 cm, FANS achieved noninferior immediate and 1-month SFR compared with mini-PCNL, while operative time was not significantly longer [6]. Although these findings support the potential value of FANS within this stone-size range, they derive from a subgroup of the broader upper urinary tract stone population and should not be interpreted as definitive evidence of superiority specifically for 2–2.5 cm renal stones.

Overall, the available randomized evidence suggests that FANS-assisted RIRS may achieve stone-free outcomes comparable to mini-PCNL in selected patients with 2–3 cm stones, with potential advantages in bleeding-related outcomes and postoperative recovery. Nevertheless, interpretation must account for overlap between individual trials and pooled analyses, differences in study populations, and the limited evidence for larger or more complex renal stones.

### 3.6. Has Modern RIRS Closed the Gap with PCNL?

The contemporary evidence suggests that the answer depends substantially on the type of RIRS being considered and the stone burden being treated.

When conventional RIRS is compared with mini-PCNL across randomized trials, mini-PCNL continues to provide superior single-session stone clearance [2]. This advantage is clinically relevant because repeated procedures impose additional anesthetic exposure, cost, inconvenience, and cumulative procedural burden. However, conventional RIRS offers advantages in bleeding-related outcomes and hospitalization [2].

The emerging evidence for suction-assisted RIRS presents a different picture. In patients with 2–3 cm renal stones, FANS-assisted RIRS has achieved stone-free outcomes comparable to mini-PCNL in recent randomized trials and meta-analyses while maintaining advantages in bleeding, postoperative pain, and length of hospital stay [4,5,6].

However, these findings remain dependent on the methodological characteristics and stone-free definitions of a relatively small number of randomized trials, and published correspondence concerning the pivotal Zeng et al. trial highlights the need for cautious interpretation and further independent validation.

These findings do not establish RIRS as a universal replacement for PCNL. The strongest current evidence for FANS relates specifically to stones measuring 2–3 cm. These results should not automatically be extrapolated to substantially larger stones, complete or complex staghorn calculi, or very high-volume stone disease, for which PCNL remains the established reference approach [1].

Instead, the available evidence supports a more selective treatment paradigm. PCNL remains particularly appropriate when stone burden is large or complex and when maximal single-session clearance is the principal therapeutic objective. For appropriately selected 2–3 cm stones, particularly when minimizing bleeding, postoperative pain, and hospitalization is important, contemporary suction-assisted RIRS represents an increasingly credible alternative. Emerging evidence suggests that this balance may be particularly favorable for stones between 2 and 2.5 cm [4,5,6]. The principal contemporary comparative studies informing the relative efficacy, safety, and clinical applicability of PCNL, mini-PCNL, conventional RIRS, and suction-assisted RIRS are summarized in Table 1. The principal clinical factors that may influence selection between PCNL and contemporary RIRS are summarized in Table 2.

## 4. Discussion

The management of renal stones larger than 2 cm is entering a period of important technological transition. PCNL remains the guideline-recommended first-line treatment and continues to provide the most reliable route to high single-session stone clearance, particularly for large-volume and complex renal calculi [1]. However, the increasing effectiveness of contemporary RIRS means that stone size alone is becoming an insufficient basis for treatment selection.

The central finding of the contemporary evidence is that the relative advantages of PCNL and RIRS depend on the outcomes considered. In randomized comparisons of conventional RIRS and mini-PCNL, mini-PCNL generally achieves a higher single-session stone-free rate, whereas RIRS is associated with reduced bleeding, fewer transfusion requirements, and shorter hospitalization [2]. These findings specifically concern mini-PCNL and should not be interpreted as a direct comparison between RIRS and standard PCNL. This distinction is clinically important because the stone-free rate should not be interpreted in isolation. A procedure that achieves greater immediate clearance may simultaneously expose the patient to greater access-related morbidity, whereas a less invasive procedure may reduce perioperative burden at the expense of a greater probability of staged or auxiliary treatment.

### 4.1. From Conventional RIRS to Technology-Assisted RIRS

An important implication of recent evidence is that RIRS should no longer be considered a single, technologically uniform procedure. Conventional flexible ureteroscopy, modern laser-assisted RIRS, and suction-assisted RIRS differ substantially in their ability to manage increasing stone burden.

Advances in laser technology have improved the efficiency of intracorporeal lithotripsy. TFL and modern Ho:YAG platforms provide effective fragmentation and dusting during ureteroscopy [3]. However, improved fragmentation alone does not solve the entire problem of treating large renal stones. As stone volume increases, the surgeon must manage not only fragmentation but also visualization, fragment evacuation, irrigation, operative duration, and intrarenal pressure. Consequently, the evolution of RIRS for larger stones depends on the integration of multiple technologies rather than on the laser platform alone.

This distinction also explains why TFL should not be positioned as an independent competitor to PCNL. TFL is an enabling technology within an endoscopic treatment strategy. The clinically relevant comparison is therefore between a percutaneous strategy and an increasingly sophisticated retrograde strategy incorporating modern lithotripsy, access, visualization, and fragment-management technologies.

### 4.2. Suction as a Potential Change in the Treatment Paradigm

The development of FANS may represent a more consequential change for large-stone RIRS than improvements in laser technology alone. Conventional dusting can generate a substantial volume of residual particulate material, and fragment extraction becomes progressively more demanding as stone burden increases. Active suction introduces the possibility of simultaneous lithotripsy and fragment evacuation and therefore addresses one of the practical limitations of conventional RIRS.

Recent randomized evidence is particularly important in this regard. In selected patients with 2–3 cm renal stones, FANS-assisted flexible ureteroscopy has produced stone-free outcomes approaching those of mini-PCNL while reducing bleeding-related morbidity and hospitalization [4,5,6]. The 2026 meta-analysis of randomized trials similarly found comparable final SFR between FANS-RIRS and mini-PCNL, with advantages for FANS-RIRS in hemoglobin reduction, hemorrhage, transfusion, postoperative pain, and hospital stay [5].

The randomized trials and the subsequent meta-analysis should not be regarded as independent bodies of evidence, because the meta-analysis synthesizes randomized trial data. The pooled findings should therefore be interpreted as a synthesis of the available randomized evidence rather than as independent corroboration of the individual trials.

These results suggest that the traditional efficacy–morbidity trade-off between RIRS and PCNL may be changing. With conventional RIRS, choosing the retrograde route often means accepting lower single-session clearance in exchange for reduced invasiveness [2]. With suction-assisted RIRS, recent evidence suggests that some of this efficacy disadvantage may be reduced, at least for selected stones within the 2–3 cm range [4,5,6].

Nevertheless, these findings should be interpreted cautiously. The evidence base for FANS remains substantially smaller than that supporting PCNL, and the strongest randomized data currently relate predominantly to stones between 2 and 3 cm [4,5,6]. It would therefore be inappropriate to extrapolate these findings directly to very large stone burdens, complete staghorn calculi, or highly complex collecting-system stones.

### 4.3. Stone Size Should Not Be Treated as a Binary Threshold

Current guidelines appropriately use stone size as a major determinant of treatment and recommend PCNL as first-line treatment for renal stones >2 cm [1]. However, the emerging evidence raises an important question regarding whether all stones above this threshold should be considered equivalent.

A 2.1 cm solitary renal pelvic stone and a 4 cm branched or staghorn calculus both satisfy the definition of a stone >2 cm, yet they represent fundamentally different procedural challenges. Stone volume, number, distribution, location, collecting-system anatomy, infundibular accessibility, density, and anticipated fragment burden may all influence the feasibility of retrograde treatment.

In a comparative study of 250 patients with renal stones measuring 2–4 cm, Cozma et al. found that PCNL achieved a higher first-session stone-free rate than flexible ureteroscopy (90.4% vs. 68%), whereas flexible ureteroscopy was associated with a shorter hospital stay; overall complication rates were not significantly different [12].

Broader pooled evidence also suggests that treatment success in this size range depends on how efficacy is defined across the entire treatment pathway. Saad et al. reported a systematic review and meta-analysis of 20 studies involving 2326 patients with 2–4 cm renal stones [13]. The pooled analysis found no statistically significant difference in overall stone-free rate between FURS and mini-PCNL (effect estimate 0.98, 95% CI 0.95–1.01; *p* = 0.133). However, FURS demonstrated a more favorable morbidity profile, with significantly shorter hospitalization, less hemoglobin reduction, lower transfusion rates, and fewer overall complications (RR 0.52, 95% CI 0.41–0.65; *p* < 0.0001) [13]. The authors therefore concluded that FURS represents a highly effective and safer alternative to mini-PCNL for selected 2–4 cm renal stones, providing comparable efficacy while reducing surgical morbidity and hospitalization. These findings nevertheless require consideration of heterogeneity among the included studies and the potential need for staged ureteroscopic treatment.

The recent randomized evidence is concentrated predominantly within the 2–3 cm stone-size range [4,5,6]. Wang et al. reported results for a 2–2.5 cm subgroup; however, subgroup findings require cautious interpretation and should not be taken as definitive evidence of a treatment advantage without consideration of the reported subgroup analysis, its statistical precision, and any interaction testing [6]. These findings reinforce the need to evaluate stone burden more granularly rather than treating all renal stones >2 cm as a homogeneous category.

The practical implication is not that the 2 cm guideline threshold should be abandoned. Rather, the threshold should remain an important starting point for decision-making while additional anatomical, procedural, and patient-specific factors determine whether a percutaneous or retrograde strategy is preferable.

### 4.4. Patient Selection Is Becoming Increasingly Important

The expanding capabilities of RIRS make appropriate patient selection increasingly important. PCNL should remain strongly favored when maximal single-session clearance is essential, particularly in patients with substantial stone burden, complex or staghorn calculi, or anatomy that would make complete retrograde treatment difficult [1].

Conversely, contemporary RIRS may be particularly attractive when avoidance of percutaneous access is desirable or when minimizing bleeding, postoperative pain, and hospitalization carries greater clinical importance [2,4,5,6]. The availability of suction-assisted technology may further strengthen this option for selected 2–3 cm stones.

Patient preference should also form part of this discussion. Some patients may prioritize the highest probability of single-session clearance despite accepting a more invasive procedure, whereas others may accept the possibility of additional treatment in exchange for avoiding percutaneous access and potentially achieving a faster postoperative recovery. Shared decision-making should therefore communicate not simply the expected SFR but also the probability of staged treatment, procedural morbidity, hospitalization, and recovery.

### 4.5. Implications for Contemporary Practice

The current evidence does not justify replacing PCNL as the reference treatment for renal stones >2 cm. The 2026 EAU guideline continues to recommend PCNL as first-line treatment for larger stones, and RIRS frequently requires staged procedures as stone burden increases [1]. This remains particularly relevant for complex and staghorn calculi.

However, the evidence also suggests that the traditional statement that “stones >2 cm require PCNL” is becoming too simplistic for contemporary practice. Modern RIRS, particularly when combined with suction-assisted access, can provide competitive outcomes for selected 2–3 cm stones while reducing several measures of perioperative morbidity [4,5,6].

The evolving treatment paradigm may therefore be better expressed as a continuum rather than a binary choice. At one end there are smaller and anatomically favorable stones for which RIRS is well established. At the other end there are very large, complex, and staghorn stones for which PCNL remains clearly favored. Between these groups lies an increasingly important intermediate population—particularly patients with noncomplex stones between approximately 2 and 3 cm—in whom either mini-PCNL or contemporary RIRS may be appropriate depending on stone characteristics, patient factors, available technology, and surgeon expertise.

### 4.6. Limitations of the Current Evidence

Several limitations should temper interpretation of the available literature. First, definitions of stone-free status vary considerably between studies. Complete absence of fragments and thresholds permitting residual fragments of 2–4 mm are not equivalent outcomes, and differences in definitions can substantially influence reported SFR.

Second, postoperative imaging is heterogeneous. Non-contrast computed tomography is more sensitive for residual fragments than ultrasonography or plain radiography, making direct comparisons between studies using different imaging protocols potentially misleading.

Third, the term RIRS encompasses increasingly heterogeneous technology. Outcomes achieved with conventional access sheaths and standard lithotripsy should not automatically be assumed to represent those achievable with suction-assisted systems, modern digital ureteroscopes, or newer laser platforms.

Fourth, much of the recent randomized evidence for FANS is concentrated within the 2–3 cm stone-size range [4,5,6]. In addition, methodological concerns regarding the pivotal Zeng et al. noninferiority trial have been raised in published correspondence, including issues related to trial design, stone-free assessment, and generalizability, with subsequent responses from the trial investigators. These considerations do not negate the randomized findings but reinforce the need for independent validation and caution when extrapolating them beyond the populations and protocols studied.

Fifth, the applicability of some comparative evidence to the target population is limited by differences in stone size and anatomical eligibility. The meta-analysis by Alharran et al. included renal stones measuring 1–3 cm; therefore, its pooled estimates should not be interpreted as outcomes specific to stones >2 cm. The randomized trial by Wang et al. enrolled patients with 2–3 cm upper urinary tract stones, and its overall findings should not be presented as exclusively renal-stone-specific unless separate renal subgroup results are available. These limitations are particularly relevant when considering renal stones >3 cm or complex stone configurations, for which direct comparative evidence remains limited [2,6].

Sixth, technological availability and surgical expertise vary considerably between institutions. Outcomes reported by high-volume endourology centers using contemporary equipment may not be immediately generalizable to all clinical environments.

For these reasons, current evidence supports expansion of the role of modern RIRS rather than wholesale replacement of PCNL. Future comparative trials should use standardized definitions of stone-free status, preferably CT-based assessment where appropriate, stratify outcomes according to stone size and complexity, and distinguish clearly between conventional and suction-assisted RIRS.

### 4.7. Future Directions

Future comparative research should move beyond stone diameter as the principal determinant of treatment selection. Although the 2 cm threshold remains clinically useful, stones of similar maximal diameter may differ substantially in total stone volume, number, distribution, location, density, and anatomical complexity. Collecting-system anatomy, including lower-pole location, infundibular accessibility, calyceal distribution, and the presence of partial or complete staghorn configuration, may substantially influence the feasibility and efficiency of a retrograde approach. Future trials should therefore stratify patients according to stone burden and anatomical complexity rather than relying predominantly on maximal stone diameter.

Treatment success should also be evaluated beyond the conventional stone-free rate. For larger renal stones, clinically relevant outcomes include the probability of achieving clearance in a single session, cumulative operative time, requirement for staged procedures or auxiliary interventions, complications, transfusion, postoperative pain, hospital stay, readmission, and time to complete treatment. A strategy associated with lower morbidity per procedure may not necessarily result in a lower overall treatment burden if multiple procedures are required. Conversely, a more invasive procedure may remain preferable when the probability of complete clearance in a single session is substantially greater. Future comparative studies should therefore report both per-procedure outcomes and cumulative outcomes across the entire treatment pathway.

The role of suction-assisted endourology requires further evaluation. Current randomized evidence for FANS-assisted RIRS is concentrated predominantly in stones measuring 2–3 cm [4,5,6]. Larger multicenter trials should determine whether these results remain reproducible in stones >3 cm and in patients with multiple, lower-pole, anatomically complex, or partial staghorn calculi. Comparative studies should also clearly distinguish conventional RIRS from suction-assisted RIRS and standard PCNL from miniaturized percutaneous techniques, because these approaches differ substantially in access, fragment evacuation, morbidity, and treatment efficiency.

Technological development will continue to influence these comparisons. Improvements in laser lithotripsy, ureteroscope design, suction-assisted access, intrarenal-pressure control, and percutaneous miniaturization may alter the relative advantages of retrograde and percutaneous approaches [14]. Future studies should evaluate these technologies as components of an integrated treatment strategy rather than assessing individual devices in isolation [15].

Artificial intelligence may eventually contribute to endoscopic stone recognition, intraoperative decision support, procedural standardization, and outcome prediction [16,17,18]. However, its present clinical role in determining the choice between PCNL and RIRS for renal stones >2 cm remains investigational. Future AI-based studies should demonstrate clinically meaningful improvements in treatment selection, procedural safety, or patient outcomes before these technologies can be incorporated into routine decision-making.

Ultimately, future research should aim to define which patients benefit most from each treatment strategy rather than attempting to establish a single universally superior procedure. Prospective trials incorporating standardized CT-based stone-free assessment, validated measures of stone burden and complexity, cumulative treatment burden, patient-reported outcomes, and cost-effectiveness may allow treatment selection to evolve from a predominantly size-based algorithm toward a more individualized approach.

## 5. Conclusions

PCNL remains the reference standard for renal stones >2 cm, particularly when stone burden is large or complex and maximal single-session clearance is the primary treatment objective. Its established efficacy and ability to manage complex and staghorn calculi mean that contemporary technological advances do not currently justify replacing PCNL as the first-line approach across this heterogeneous patient population.

However, the traditional concept that all renal stones >2 cm should automatically be managed by PCNL is becoming increasingly nuanced. Modern RIRS has evolved substantially through improvements in flexible endoscopy, laser lithotripsy, fragment management, and particularly suction-assisted access. Recent randomized evidence suggests that FANS-assisted RIRS can achieve stone-free outcomes comparable to mini-PCNL in appropriately selected patients with 2–3 cm stones while offering advantages in bleeding-related morbidity, postoperative pain, and hospitalization.

The contemporary question is therefore no longer simply whether PCNL or RIRS is superior. Treatment should instead be individualized according to stone size, volume and complexity, collecting-system anatomy, the importance of single-session clearance, patient comorbidity and preference, available technology, and surgeon expertise. PCNL remains the benchmark for complex and high-volume stone disease, while technology-assisted RIRS is emerging as a credible alternative within the increasingly important 2–3 cm treatment space. Future randomized studies using standardized stone-free definitions and imaging protocols will be essential to define more precisely where the boundary between these approaches should lie.

## Figures and Tables

**Table 1:** Key Contemporary Comparative Evidence for PCNL and RIRS.

Study	Design and Population	Comparison	Principal Efficacy Finding	SFR Definition/ Imaging/Timing	Safety and Recovery Findings	Clinical Interpretation
RIRS vs. mini-PCNL meta-analysis, 2026 [2]	Meta-analysis; 30 RCTs; 4173 patients; renal stones 1–3 cm	Conventional RIRS vs. mini-PCNL	Mini-PCNL achieved significantly higher single-session SFR	Definitions, imaging modalities, and assessment timing varied across the included RCTs; single-session SFR was the principal pooled efficacy outcome	RIRS: less Hb reduction, fewer transfusions, and shorter hospitalization; more auxiliary procedures	Mini-PCNL retains an advantage in single-session clearance, whereas RIRS reduces access-related morbidity
Zeng et al., 2026 [4]	Randomized noninferiority trial; 720 patients; 12 centers; renal stones 2–3 cm	FANS-assisted fURS vs. mini-PCNL	FANS-fURS noninferior for immediate SFR; no significant difference in 3-month SFR	Immediate and 3-month SFR were assessed; SFR definition and imaging modality should be interpreted according to the original trial protocol	FANS: longer operative time, lower transfusion rate, shorter hospitalization, and greater QoL improvement	Large multicenter randomized evidence supporting FANS-assisted RIRS in selected patients with 2–3 cm renal stones; interpretation remains dependent on the noninferiority design, SFR methodology, and generalizability.
Li et al., 2026 [5]	Meta-analysis; 3 RCTs; 1020 patients; renal stones 2–3 cm	FANS-RIRS vs. mini-PCNL	No significant difference in final SFR	SFR definitions, imaging modalities, and assessment timing varied across the constituent RCTs; final SFR was evaluated as a pooled outcome	FANS-RIRS: less Hb loss, hemorrhage, transfusion, postoperative pain, and shorter hospitalization	Supports FANS-RIRS as an alternative for selected 2–3 cm renal stones
Wang et al., 2026 [6]	RCT; 326 patients; upper urinary tract stones 2–3 cm	FANS-assisted fURS vs. mini-PCNL	Comparable immediate and 1-month SFR	SFR was assessed immediately after treatment and at 1 month; the reported assessment time points are presented here because uniform imaging and residual-fragment criteria could not be established from the available study information.	FANS: less Hb loss and shorter hospitalization; longer overall operative time	Supports consideration of FANS for selected 2–3 cm upper urinary tract stones; the 2–2.5 cm subgroup findings should be interpreted cautiously

**Table 2:** Clinical Factors Potentially Favoring PCNL or Contemporary RIRS.

Clinical Consideration	PCNL/Mini-PCNL	Contemporary RIRS
Renal stone >2 cm	Guideline-recommended first-line approach [1]	Alternative in appropriately selected patients
Stone burden 2–3 cm	Established and effective approach	Increasingly supported, particularly with FANS [4,5,6]
Very large stone burden	Favored	Greater likelihood of prolonged or staged treatment
Partial/complete staghorn calculus	Favored	Generally not preferred as primary treatment
Maximal single-session clearance required	Favored [2]	Higher likelihood of auxiliary/staged treatment with conventional RIRS [2]
Minimizing bleeding	Greater access-related bleeding burden	Favored, particularly with FANS [2,4,5,6]
Short hospitalization prioritized	Generally less favorable	Favored [2,4,5,6]
Avoidance of percutaneous renal access	Not applicable	Favored
Availability of suction/FANS technology	Not applicable	May improve applicability to 2–3 cm stones [4,5,6]
Stones 2–2.5 cm with appropriate anatomy	Effective	Potential indication for FANS-assisted RIRS; subgroup evidence should be interpreted cautiously [6]
Complex/high-volume stone disease	Favored	Current evidence insufficient to replace PCNL

## Data Availability

No new data were created or analyzed in this study. Data sharing is not applicable to this article.

## References

[1] (2026). EAU Guidelines. Edn. Presented at the EAU Annual Congress London 2026.

[2] Alharran A.M., Albloushi M.H., Ali A.M., Aldousari A.H., Gadelmawla A.F., Taleb A.H., Alaradi H. (2026). Retrograde intrarenal surgery versus miniaturized percutaneous nephrolithotomy for renal calculi: A GRADE-assessed meta-analysis of randomized controlled trials with trial sequential analysis. World J. Urol..

[3] Ulvik Ø., Æsøy M.S., Juliebø-Jones P., Gjengstø P., Beisland C. (2022). Thulium Fibre Laser versus Holmium:YAG for Ureteroscopic Lithotripsy: Outcomes from a Prospective Randomised Clinical Trial. Eur. Urol..

[4] Zeng G., Jiang K., Liu S., Wu R., Duan X., Chai C.A., Gökce M.I., Gadzhiev N., Kalathia J., Wang K. (2026). Flexible Ureteroscopy with a Flexible and Navigable Suction Ureteral Access Sheath Versus Mini-Percutaneous Nephrolithotomy for Treatment of 2–3 cm Renal Stones: An International, Multicenter, Randomized, Noninferiority Trial. Eur. Urol..

[5] Li Y., Yao Z., Zhou Z., Wang H., Zhang Y., Zong H. (2026). Efficacy and safety of retrograde intrarenal surgery with a flexible and navigable suction sheath (FANS-RIRS) versus mini-percutaneous nephrolithotomy (mPCNL) for 2–3 cm renal stones: A systematic review and meta-analysis of randomized controlled trials. World J. Urol..

[6] Wang R., Qi X., Li C., Huang J., Xie L., Liu L., Xie H., Liu C. (2026). F-URS with FANS versus mPCNL for 2–3 cm UUTS: A RCT with a subgroup analysis of 2–2.5 cm stones. BJU Int..

[7] Wishahi M., El Feel A., Elkhouly A., Fahmy A., Roshdy M., Elbaz A.G., I Kamel A., Badawy M., Elesaily K., Eldahshan S. (2023). Concerns about stone free rate and procedure events of percutaneous nephrolithotripsy (PCNL) for 2–4 cm kidney stones by standard-PCNL vs mini-PCNL—Comparative randomised study. BMC Urol..

[8] Adhoni M.Z.U., Pooley E., Byrnes K., Tzelves L., Juliebø-Jones P., Gauhar V., Yuen S.K.K., Traxer O., Somani B. (2026). Suction in mini-percutaneous nephrolithotomy: A meta-analysis from the European Association of Urology Section of Endourology. BJU Int..

[9] Hinojosa-Gonzalez D.E., Saffati G., Kronstedt S., Rodriguez C., La T., Link R.E., Mayer W.A. (2025). Endourological Management of Renal Stones: A Systematic Review, Bayesian Network Meta-analysis and Meta-regression. Urology.

[10] Kassem A., Torad H., Essam A., Abdel Hamid M., Zamel S., Elkady A. (2026). Retrograde intra renal surgery (RIRS) versus percutaneous nephrolithotomy (PNL) as a primary treatment for large renal stones: A prospective randomized controlled trial. Arab J. Urol..

[11] Riveros C.A., Chalfant V., Melchart T., Singh G., Forero A.M., Ledesma B., Harnett S., A Stec A., Feloney M., Delto J.C. (2022). Does Moses technology improve the efficiency and outcomes of standard holmium laser lithotripsy? A systematic review and meta-analysis. Cent. Eur. J. Urol..

[12] Cozma C., Georgescu D.A., Geavlete P., Popescu R.I., Geavlete B. (2023). Comparison between Retrograde Flexible Ureteroscopy and Percutaneous Nephrolithotomy for the Treatment of Renal Stones of 2–4 cm. Medicina.

[13] Saad M.A., Nasr A.M., Keshk T.F., Qudaih R.F., Abdelaziz A.S.A., Said M. (2026). Flexible Ureteroscopy Versus Minipercutaneous Nephrolithotomy for 2 to 4 cm Renal Stones: A Systematic Review and Meta-Analysis. Surg. Innov..

[14] Yuen S.K.K., Zhong W., Chan Y.S., Castellani D., Bhojani N., Agarwal M.S., Tokas T., Croghan S., Jung H., Herrmann T. (2025). Current utility, instruments, and future directions for intra-renal pressure management during ureteroscopy: Scoping review by global research in intra-renal pressure collaborative group initiative. Ther. Adv. Urol..

[15] Spinos T., Nowak L., Somani B.K., Omar M., Tsaturyan A., Kallidonis P., Liatsikos E., Pietropaolo A. (2026). Flexible ureteroscopy with integrated suction, a useful innovation: A systematic review by the YAU and EAU endourology sections. Curr. Opin. Urol..

[16] Nedbal C., Cacciamani G., De Backer P., Ghnatios C., Khene Z.E., Kronenberg P., Tefik T., Turney B., Traxer O., Somani B.K. (2025). Artificial Intelligence for Endoscopic Stone Surgery: What’s Next? An Overview from the European Association of Urology Section of Endourology. Eur. Urol. Focus..

[17] Wang C., Liang H., Chen H., Khan R., Shen D., Liu H., Shen D., Wang W., Liu J., Panthier F. (2025). Clinical validation of an AI-assisted system for real-time kidney stone detection during flexible ureteroscopic surgery. npj Digit Med..

[18] Morozov A., Matkovskiy I., Somani B., Baniel J., Traxer O., Lifshitz D., Ali S., Erlich Y., Golan S., Pushkar D. (2025). Intraoperative use of artificial intelligence (AI) during endoscopic lithotripsy: A systematic review from EAU endourology. World J. Urol..

